# Global transcriptome changes in perennial ryegrass during early infection by pink snow mould

**DOI:** 10.1038/srep28702

**Published:** 2016-06-27

**Authors:** Mallikarjuna Rao Kovi, Mohamed Abdelhalim, Anil Kunapareddy, Åshild Ergon, Anne Marte Tronsmo, May Bente Brurberg, Ingerd Skow Hofgaard, Torben Asp, Odd Arne Rognli

**Affiliations:** 1 Department of Plant Sciences, Norwegian University of Life Sciences, NO-1432 Ås, Norway; 2 Division of Biotechnology and Plant Health, Norwegian Institute of Bioeconomy Research (NIBIO), NO-1432 Ås, Norway; 3 Department of Molecular Biology and Genetics, Aarhus University, Slagelse, Denmark

## Abstract

Lack of resistance to pink snow mould (*Microdochium nivale*) is a major constraint for adaptation of perennial ryegrass (*Lolium perenne* L.) to continental regions with long-lasting snow cover at higher latitudes. Almost all investigations of genetic variation in resistance have been performed using cold acclimated plants. However, there may be variation in resistance mechanisms that are functioning independently of cold acclimation. In this study our aim was to identify candidate genes involved in such resistance mechanisms. We first characterized variation in resistance to *M. nivale* among non-acclimated genotypes from the Norwegian cultivar ‘Fagerlin’ based on relative regrowth and fungal quantification by real-time qPCR. One resistant and one susceptible genotype were selected for transcriptome analysis using paired-end sequencing by Illumina Hiseq 2000. Transcriptome profiles, GO enrichment and KEGG pathway analysis indicate that defense response related genes are differentially expressed between the resistant and the susceptible genotype. A significant up-regulation of defense related genes, as well as genes involved in cell wall cellulose metabolic processes and aryl-alcohol dehydrogenase (NADP+) activity, was observed in the resistant genotype. The candidate genes identified in this study might be potential molecular marker resources for breeding perennial ryegrass cultivars with improved resistance to pink snow mould.

Perennial ryegrass (*Lolium perenne* L.,) belongs to the Poaceae family. It is a diploid species (2n = 2x = 14) native to Europe, Asia and Northern Africa^1^. It is an important forage grass in the temperate regions of the world because of its high forage quality and yield. Out of 52 million ha of grasslands in Europe, 23% is cultivated with *Lolium* species, with perennial ryegrass being the most widespread. Perennial ryegrass has low resistance against pink snow mould, however, tetraploid cultivars have better resistance than diploid and turf cultivars^1^.

Winter injury is regarded as a serious constraint for the production of winter cereals and grasses at northern latitudes^2^,^3^. The fungus *Microdochium nivale* (Fr.) Samuels & Hallet is considered to be the most widespread cause of biotic winter injury in these crops^4^. It is an opportunistic species causing pink snow mould on winter cereals, turf and forage grasses at low temperatures, with or without a snow cover. High humidity and constant low temperatures under snow cover are highly favourable for the development of pink snow mould^5^,^6^.

Resistance to pink snow mould is enhanced by cold acclimation^2^,^3^,^4^. During this process the plant undergoes numerous physiological and bio-chemical changes which are essential for winter survival^7^. Some of these changes are also thought to increase resistance to diseases, e.g. cellular dehydration and accumulation of defense-related proteins and fructans^8^. Genetic variation in cold-induced resistance to pink snow mould in triticale has been shown to be associated with changes in physical and chemical properties of the leaf surface and cell walls^9^, and with photosynthetic acclimation and peroxidase activity^10^.

Previous studies on pink snow mould resistance has almost exclusively been performed on cold acclimated plants. However, some genetic variation in resistance is also present in non-acclimated winter wheat^11^, and this resistance may be masked when testing cold acclimated plants. Inherent resistance that is independent of cold acclimation may be more specific to *M. nivale* than cold-induced disease resistance, and is likely to be important for preventing diseases caused by *M. nivale* during the growing season, such as microdochium patch and leaf blotch. It may also be increasingly important with the predicited climate change^12^. The projected climatic conditions^12^ during fall may not allow plants to go through this process, and plants may therefore be covered with snow before they are cold hardened. Our overall aim was to identify genotypes that are able to resist/ tolerate a snow mold infection without cold hardening before exposure to the winter stress factors.

The use of molecular techniques for precise quantification of the fungal biomass in infected plants can facilitate the selection of resistant genotypes^13^. Usage of real-time PCR in quantification of plant pathogen infestation has increased in the last two decades. It is quicker, more specific and sensitive compared to traditional methods based on symptom assessment or plant dry weight^14^. Therefore, elongation factor 1a (*EF-1a*) gene was used in our study for accurate quantification of *M. nivale* DNA during snow mould infestation. The *EF-1a* gene has formerly been used to recognize *M. nivale* and *M. majus* as separate species^15^. The *EF-1a* has also been used to study the genetic variation among isolates^16^. Additionally, competitive PCR methods have also been developed for *M. nivale* and *M. majus* quantification in infected tissues^17^.

The use of whole transcriptome sequencing (RNA-seq) is an important tool in the analysis of complex plant responses^18^, and provides a more comprehensive understanding of transcription initiation sites, improved detection of alternative splicing events and the detection of gene fusion transcripts^19^. The technology is able to handle *de novo* sequencing of large genomes, revealing individual genome differences within the same species and quantify gene expression^20^. In particular, it now enables global transcriptome studies to be performed in non-model species that have lacked many of the array based assays that are successfully used to study gene expression in the model species.

In the present work, we have taken advantage of high throughput RNA-seq to study the global transcriptome changes in perennial ryegrass leaves during early infection by pink snow mould, more spesifically after four days incubation under artifical snow cover (Supplementary Fig. S1). The aim of the present study was to identify molecular responses involved in snow mould resistance independent of cold acclimation in perennial ryegrass.

## Results

### Snow mould resistance test

**Figure 1:**
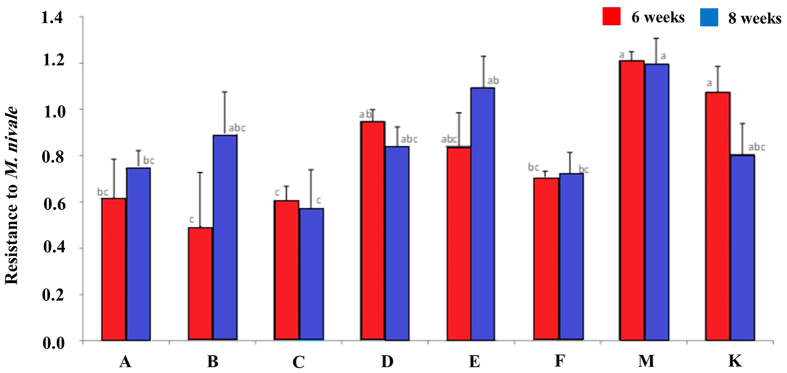
Resistance to *M. nivale* in 8 genotypes, measured as relative regrowth (dry weight of inoculated plants divided by dry weight of non-inoculated plants) after 6 and 8 weeks incubation under artificial snow cover followed by two weeks of regrowth. Error bars indicate standard errors of the mean, and bars marked with different letters are significantly different (P < 0.05).

Eight genotypes of *L. perenne* cv. Fagerlin were randomly selected and evaluated for resistance to *M. nivale*. There was a significant effect of genotype on resistance, measured as relative regrowth after inoculation and incubation under artificial snow cover for 6 and 8 weeks (Fig. 1). The differences between genotypes were most pronounced after 6 weeks. Overall, genotype M had the highest relative regrowth, while there were small differences among the other genotypes (Fig. 1). Based on the results of this resistance test we selected two genotypes for studying global changes in the transcriptome during snow mould infection; genotype F as a “susceptible” genotype (later termed S) and genotype M as a “resistant” genotype (later termed R).

### Quantification of *M. nivale* DNA

**Figure 2:**
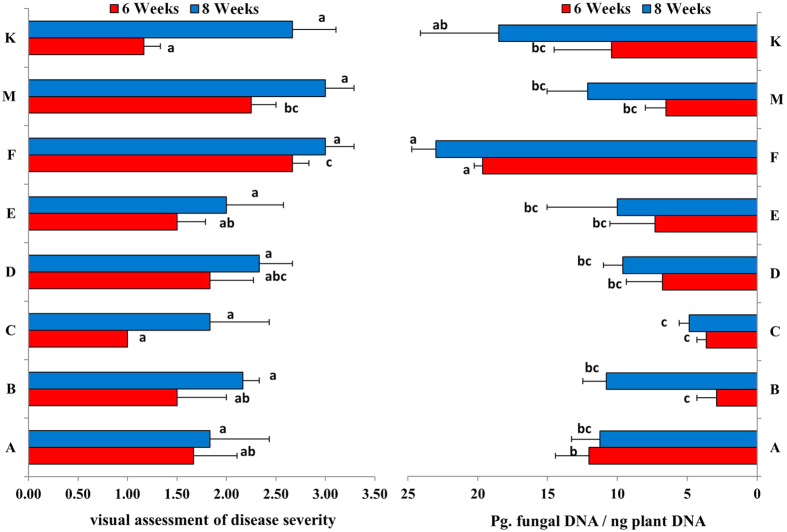
Visual assessment of disease symptoms; scale 0 (no symptoms) to 4 (severely diseased), and amount of *M. nivale* (isolate 200231) DNA (pg. fungal DNA/ng plant DNA) in 8 genotypes of *L. perenne* cv. Fagerlin after 6 and 8 weeks of inoculation. Error bars indicate standard errors of the mean, and bars marked with different letters are significantly different (P < 0.05).

Visual assessment of disease severity and quantification of *M. nivale* DNA in leaf and stem tissue of the eight genotypes showed that genotypes with severe symptoms of injury (such as genotype F) contained significantly more fungal DNA than the other genotypes, especially after 6 weeks of incubation (Fig. 2). Genotypes that contained most fungal DNA, e.g. genotype F, were also most symptomatic based on visual scoring (Fig. 2). There was a significant positive correlation (r = 0.489, P ≤ 0.05) between disease severity and the amount of *M. nivale* DNA, indicating the genotypes containing most fungal DNA also showed most disease symptoms (Supplementary Table S1). The correlations between relative regrowth and the amount of *M. nivale* DNA, and relative regrowth and visual scoring of symptoms were non-significant. Tissue samples collected from the “resistant” genotype M(R) and the “susceptible” genotype F(S) showed small differences in the amount of fungal DNA 1 day after inoculation, but 4 days after inoculation the differences were more significant in the “susceptible” genotype F(S) (Supplementary Fig. S2).

### *De novo* based susceptible (S) and resistant (R) transcriptome assemblies

A total of 178 million reads and 165 million reads of 100 bp were generated for the S and R genotypes, respectively (Table 1). Separate transcriptome assemblies were generated for each genotype using all their respective reads. The *de novo* assembly yielded 261,978 contigs for the S genotype, with N50 of 1,784 bp, and 188,355 contigs for the R genotype with N50 of 1,672 bp (Table 1). The longest assembled contigs in the S and R genotype were 17,632 and 12,882 bp, respectively. To estimate the quality of the assemblies, we compared them to the *Brachypodium distachyon* coding sequence consisting of 31,029 entries. There were 27,135 *B. distachyon* sequences (87.45 percent) that had a significant hit in the S transcriptome assembly and 27,399 (88.30 percent) that had a significant hit in the R transcriptome assembly. Further, we used the CEGMA pipeline^21^ to evaluate the completeness of our assemblies. The percentage of complete core eukaryotic genes (CEGs) in R and S assemblies are 82.66 and 93.95, respectively, and the percentage of partially complete CEGs ranged from 90.73 to 98.79 (Table 2). The average number of orthologs per CEG in the R and S assemblies is 3.72 and 3.83, respectively, and the percentage of detected CEGs that had more than one ortholog was 96.1 and 97.0, respectively.

### Differentially expressed transcripts detected by *de novo* and reference based methods

**Figure 3:**
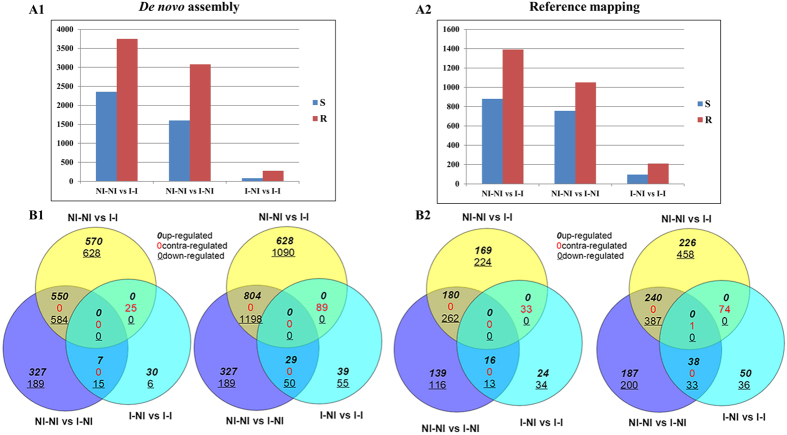
The number of differentially expressed transcripts identified using the *de novo* assembly method (**A1**) and the reference (inbred L. perenne transcriptome) based assembly method (**A2**) with FDR < 0.05. Venn diagrams showing the number of up-, down- and contra-regulated transcripts that were common and specific for the pairwise comparisons using the *de novo* assembly (**B1**) and the reference based assembly (**B2**). Contra-regulated transcripts are defined as transcripts upregulated in one condition, but downregulated in other condition. R; resistant genotype, S; susceptible genotype. NI-NI; non-inoculated and non-incubated plants, I-I; inoculated and incubated plants after 4 days of incubation, I-NI; non-inoculated and incubated plants after 4 days of incubation.

The expression levels of each assembled transcript were estimated at three different treatments, i.e. (1: Non-inoculated and non-incubated plants (NI-NI) (control plants kept in ambient temperature), 2: Non-inoculated plants incubated under artificial snow-cover for four days (I-NI), and 3: Inoculated plants that were incubated under artificial snow-cover for four days (I-I) (Artificial snow cover created by covering the plants with moist cellulose tissue paper and black plastic sheets in a cold chamber at 2 °C in darkness. The reads from each sample were mapped onto their respective genotype specific (S and R) *de novo* assemblies and to the reference inbred *L. perenne* transcriptome^22^. In the case of each sample, more than 83–90 percent of the reads mapped onto the assembled transcripts. Using the genotype-specific assemblies in a series of pairwise comparisons between samples, 2,354 and 3,748 differentially expressed transcripts were identified with false discovery rates (FDR) < 0.05 between NI-NI and I-I samples; 1,602 and 3,080 between NI-NI and I-NI samples; and 83 and 275 between I-NI and I-I samples in the S and the R genotype, respectively, with several up-, down- and contra-regulated transcripts (Fig. 3A1,B1). When using the reference based assembly mapping, 880 and 1,391 differentially expressed transcripts were identified between NI-NI and I-I samples; 755 and 1,050 between NI-NI and I-NI samples; and 95 and 210 between I-NI and I-I samples in the S and the R genotype, respectively, with several up-, down- and contra-regulated transcripts (Fig. 3A2,B2).

**Figure 4:**
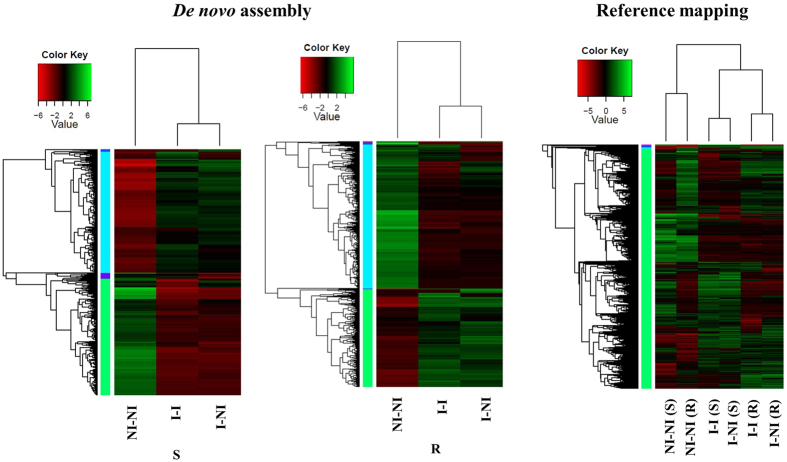
Heat maps of differentially expressed genes detected using *de novo* assemblies and reference based assembly for each genotype and grouped according to their expression patterns. X-axis represents the experimental conditions. R; resistant genotype, S; susceptible genotype. NI-NI; non- inoculated and non- incubated plants, I-I; inoculated and incubated plants after 4 days of incubation, I-NI; non-inoculated and incubated plants after 4 days of incubation.

In addition, heat maps were generated for each genotype based on the differential expression data from edgeR in order to determine the sample relationships (Fig. 4). A clear separation was seen between non-incubated (NI) and incubated (I) samples in both genotypes, whereas incubated inoculated (I-I) and incubated non-inoculated (I-NI) grouped together (Fig. 4). Even the expression data generated from reference based mapping clearly differentiated between incubated and non-incubated samples. Both S and R incubated grouped together and were separated from non-incubated samples (Fig. 4).

### Annotation and GO of differentially expressed transcripts

**Figure 5:**
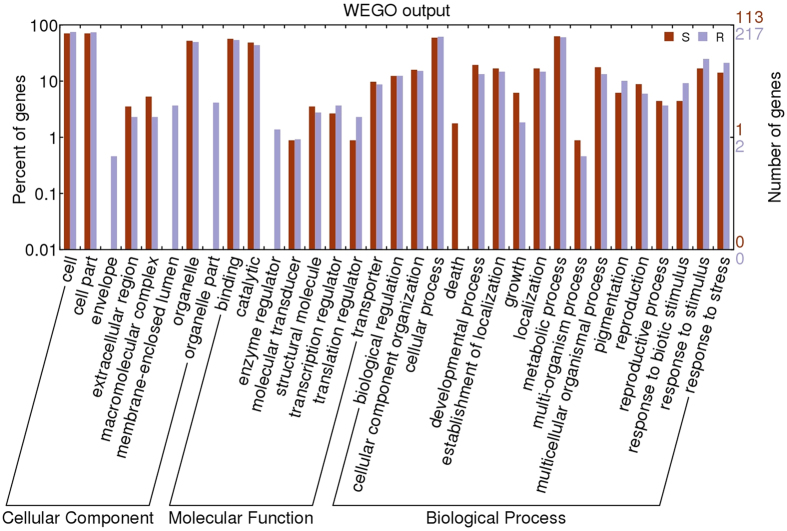
Gene ontology classifications of differentially expressed genes observed during pairwise comparisons of non-inoculated incubated (I-NI) and incubated inoculated (I-I) within resistant (R) and within susceptible (S) genotypes generated by WEGO tool http://wego.genomics.org.cn/cgi-bin/wego/index.pl) generated automatically by the web histogram tool WEGO (http://wego.genomics.org.cn/cgi-bin/wego/index.pl) using the newest GO archive provided.( The results are summarized in three main GO categories: cellular component, molecular function and biological process. The right y-axis indicates the number of genes in a category. The left y-axis indicates the percentage of a specific category of genes in that main category.

Approximately 75 percent of the differentially expressed transcripts had blast hits to the Viridiplantae database extracted from NCBI. The top hit species are *Brachypodium distachyon* followed by *Hordeum vulgare*, which are most closely related to *L. perenne*. Among the transcripts with blast hits, 40–52 percent of the differentially expressed transcripts were annotated using Blast2GO^23^. Putative descriptions and functions were assigned to the transcripts predominantly based on annotations from *H. vulgare* and *B. distachyon* and *Arabidopsis thaliana*. Gene Ontology classifications of DEGs at I-NI vs. I-I conditions in genotypes R and S were generated using WEGO^24^. The results are summarized in three main GO categories: cellular component, molecular function and biological process (Fig. 5). Comparisons of the functional categories of genotype R with those of genotype S reveal differences in terms of the biological processes. DEGs responses to stress, biotic stimulus were highly represented in the R genotype, while DEGs response to death is only seen in S genotype

**Figure 6:**
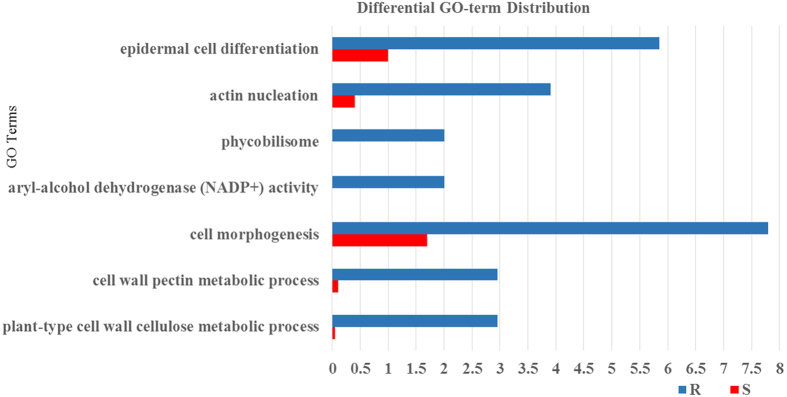
Annotation differences between resistant (R) and susceptible (S) genotypes detected by Fischer’s exact test.

Fisher’s exact test from Blast2GO^23^ was used for GO enrichment analysis between R and S to determine if any gene ontology (GO) terms were over- or under-represented in the various sets of differentially expressed transcripts. A total of seven GO terms were enriched when comparing the differentially expressed transcript sets from I-NI vs. I-I conditions of the two genotypes (Fig. 6, Supplementary Table S4). Out of these, five were overrepresented in the R genotype, in terms related to cell wall cellulose metabolic process, cell wall pectin metabolic process, cell morphogenesis, actin nucleation and organelle epidermal cell differentiation. Transcripts assigned to aryl-alcohol dehydrogenase (NADP+) activity and phycobilisome were present only in R genotype.

**Figure 7:**
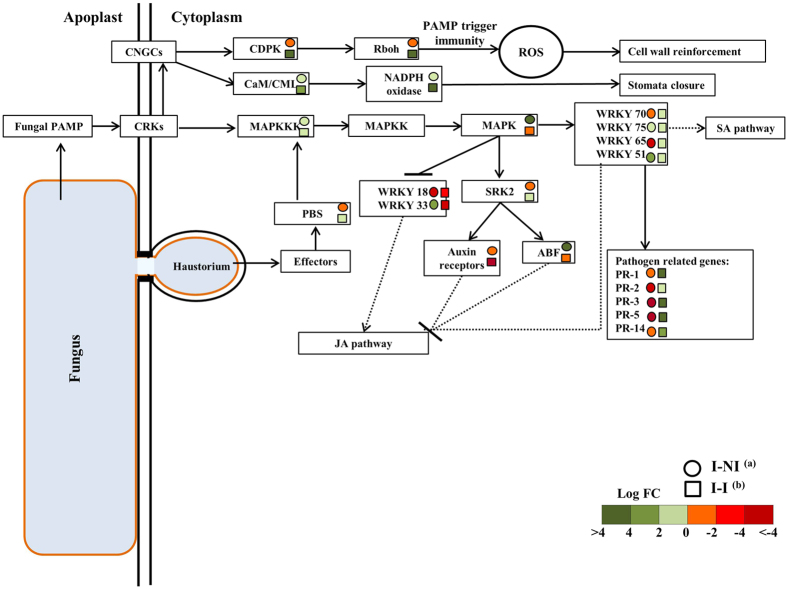
Hypothetical modules for plant-pathogen interaction after 4 days of incubation with snow mould pathogen *M. nivale* derived by KEGG plant-pathogen interaction pathway (http://www.genome.jp/kegg/) and network of WRKY transcription factors (Eulgem & Somssich (2007). Red color indicates down regulated genes and green color indicates up-regulated genes. Intensity of the colors indicates the fold change. Circle represents the fold change under non-inoculated and incubated, condition (I-NI) ^a^. The square represents the fold change under inoculated, incubated (I-I) ^b^ conditions. The recognition of pathogen-associated molecular pattern (PAMP) initiate PAMP trigger immunity via the activation of cysteine-rich receptor-like protein kinase (CRK), cyclic nucleotide gated channel (CNGC), calcium-dependent protein kinase (CDPK), respiratory burst oxidase homolog (Rboh), calcium-binding protein CML (CaM/CML), and NADPH oxidase. The activation of PAMP trigger immunity initiate the production of reactive oxygen species (ROS), which might activate the plant hypersensitive response (HR), cell wall reinforcement, as well as stomata closure. Defense responses are also instigated upon recognition of the fungal effectors in the host cell by serine/threonine-protein kinase PBS (PBS) and the activation of MAP kinase cascades such as mitogen-activated protein kinase kinase kinase (MAPKKK), mitogen-activated protein kinase kinase (MAPKK), and mitogen-activated protein kinase (MAPK). Effectors triggered immunity (ETI) initiate the production of several pathogen related proteins such as PR-1, β-1,3-glucanase (PR-2), chitinase II/V (PR-3), thaumatin-like (PR-5), and lipid-transfer protein (PR-14). Both PAMP triggered immunity and effectors triggered immunity alternate the production of salicylic acid (SA) and jasmonic acid (JA) by the action of distinct transcription factors WRKY such as WRKY 75, WRKY 70, WRKY 18, and WRKY 33. Pathogen-triggered SA signaling also by the activation of serine/threonine-protein kinase2 (SRK2), auxin receptors, and abscisic acid responsive element binding factor (ABF).

Several genes involved in the initiation of pathogen-associated molecular pattern (PAMP) immunity, like cysteine-rich receptor-like protein kinase (CRK), cyclic nucleotide gated channel (CNGC), calcium-dependent protein kinase (CDPK), respiratory burst oxidase homolog (Rboh), calcium-binding protein CML (CaM/CML), and NADPH oxidase were detected in these studies. Several pathogen related genes like *PR1*, *β-1,3-Glucanase* (*PR2*), *chitinase II/V (PR3)*, *thaumatin-like (PR5)*, and lipid-transfer protein (PR14) are upregulated in genotype R compared with genotype S under I-I conditions (Table 3, Supplementary Table S5). We also found several potential pathogen resistance candidate genes like *chitinase V*, lipid transfer protein, serine-glyoxylate aminotransferase and *WRKY 75* highly upregulated in the R genotype under I-NI treatment compared with the I-I treatment (Table 3). All potential candidate genes involved in the response of *L. perenne* to inoculation with *M. nivale* are listed in Table 3 with homologues in *A. thaliana* and *B. distachyon*. A hypothetical model for gene regulation in the plant-pathogen interaction pathway after four days of incubation with the pink snow mould pathogen, based on the pathogen related DEGs identified in this study, is presented in Fig. 7.

Furthermore, the KEGG database (http://www.genome.jp/kegg/) was used to detect different pathways in response to *M. nivale* in the S and R genotypes. Blast to the KEGG database showed that 5009 DEGs were involved in 135 pathways (Supplementary Table S2). Pathways with highest representation among the genes were involved in purine metabolism (5.19 percent, 260 genes), biosynthesis of antibiotics (5.09 percent, 255 genes), thiamine metabolism (4.25 percent, 213 genes), starch and sucrose metabolism (2.91 percent, 146 genes) and aminobenzoate degradation (2.61 percent, 131 genes).

### Validation of transcripts by real-time PCR

In order to validate the expression profiling by Illumina sequencing, the expression levels of six genes, including two *chitinase* genes, three pathogen-related genes and one WRKY family gene were further analysed by qRT-PCR. All the genes showed differential expression levels between S and R genotypes. Correlation analysis was performed between the RNA-seq and qPCR log transformed data for each gene. Among the six genes, four genes (*WRKY*, *CHI5*, *PR1* and *THI*) were highly correlated (r^2^ value in range of 0.82 to 0.97), while two genes (*CHI2*, *PR5*) were not well correlated (r^2^ values are 0.52 and 0.39) (Supplementary Fig. S3).

## Discussion

A significant positive correlation was found between the amount of *M. nivale* DNA in leaf samples and visual assessment of disease severity after 6 weeks of incubation under artificial snow cover. After 8 weeks of incubation, disease severity was similar across genotypes, while significant differences in the quantity of *M. nivale* DNA were detected. The plants incubated for a longer period (8 weeks) had a higher amount of fungal DNA than the plants incubated for 6 weeks, which is in agreement with other studies reporting that longer incubation period increases snow mould infestation even in resistant genotypes^25^,^26^. In general, neither visual assessment of disease severity, nor the amount of *M. nivale* DNA in leaves were good indicators of snow mould resistance in *L. perenne*. In the present study, plant regrowth, after inoculation with *M. nivale* and incubation for several weeks, was not correlated with fungal biomass nor disease severity. Some genotypes such as M and K showed severe symptoms on their leaf tissues, but still had good regrowth, possibly because the lower stem was not infected. On the other hand, genotype C had a poor regrowth despite limited symptoms and *M. nivale* DNA detected in the leaves (Fig. 2, Supplementary Table S1).

A qPCR test could in principle be a useful method for breeders in the selection of snow mould resistant materials. For such a test to be useful, it should be based on infestation of the lower stem tissue of the plants. More importantly, the application of quantitative real-time PCR will facilitate the detection of latent infections and early diagnosis of disease. For such purposes the test described in this study would need thorough validation with respect to sensitivity and specificity.

High throughput sequencing capabilities have made the process of assembling a transcriptome easier, even for non-model organisms without a reference genome. But the quality of a transcriptome assembly must be good enough to capture the most comprehensive catalogue of transcripts and their variations, and to carry out further transcriptomic experiments^27^. The CEGMA analysis (Table 2) showed high coverage of ultra-conserved CEGs in the assemblies of the S and R genotypes, demonstrating their completeness in terms of gene content. However, a common question is whether reference based assembly gives better results than a *de novo* based assembly.

In this study we detected a larger number of differentially expressed transcripts in a pairwise comparisons between NI-NI and I-I; NI-NI and I-NI conditions, than the pairwise comparison between I-NI and I-I condition both by *de novo* and reference based assembly mapping (Fig. 3). It was expected that there would be a larger number of transcripts differentially expressed when plants were transferred from growth (non-incubation) conditions at 20–22 °C and 18 hours of light, to incubation conditions at 2 °C and darkness due to the significant changes in temperature and light. This was seen both for the susceptible (S) and the resistant (R) genotypes, as several differentially expressed transcripts involved in rapid responses to cold stress and light, in addition to abiotic stress related transcripts were detected. On the other hand, few differentially expressed transcripts were observed between treatments I-NI and I-I in both genotypes. The annotation results of the detected transcripts between I-NI and I-I conditions in both *de novo* and reference based mapping identified similar genes involved in biotic stress, immune response and cell death. This shows the potential of *de novo* method in capturing the essential transcripts even in the absence of reference genome, which also has been demonstrated in raspberry studies^28^.

To our knowledge, this is the first transcriptome study using RNA-seq to understand the response of *L. perenne* to the early infection of pink snow mould (*M. nivale*). Several of the differentially regulated genes such as disease related proteins, calmodulin binding proteins, lipid transfer proteins, and flavonoid biosynthesis (Table 3, Supplementary Table S5) detected in the R and S genotypes between the I-NI and I-I conditions are involved in different defense-response mechanisms. The R genotype showed higher expression levels of several pathogenesis related genes such as *PR1*, *PR2*, *PR3*, *PR5*, *PR13* and *PR14*. These results are similar to those from winter wheat, where snow mould resistance was associated with the accumulation of PR1a, PR2, PR5, and PR14^29^. The higher expression levels of these PR-proteins are often considered as markers for activation of the salicylic acid (SA) signalling pathway^25^,^29^,^30^,^31^. Pociecha *et al*.^31^ also demonstrated that resistant genotypes of *Festulolium* are characterized by high SA concentrations during snow mould infection. PR proteins seem to be part of a larger set of SA and jasmonic acid (JA)-dependent defense responses in which each PR protein may contribute differently to the snow mould infection. For instance the *A. thaliana* mutant *ein2*, which is defective in ethylene/JA signalling, showed low expression level of *PR12*, *PR3* and *PR4* and high susceptibility to *B. cinerea* (necrotrophic pathogens). Conversely, the salicylic acid induction–deficient mutants of Arabidopsis expressed *PR2* and *PR5* and accumulated high levels of camalexin after pathogen inoculation^32^.

WRKY proteins, another important defense related group of proteins, constitutes a superfamily of transcription factors, involved in the regulation of different physiological platforms in plants, including pathogen defense, trichome development and senescence. In this study, *WRKY65*, *WRKY70* and *WRKY75* were upregulated after inoculation with snow mould (Table 3). It is also reported that WRKY transcription factors contributed to the defense against *Pseudomonas syringae* in tomato and play a partially conserved role in basal defense in tomato and Arabidopsis^33^.

The plant immunity system consists of two main levels^34^. The first level is based on the perception of pathogen-associated molecular patterns (PAMPs), which activates the PAMP-triggered immunity pathway (PTI). The second level is the recognition of pathogen effectors, which activates pathogen related PR genes in a process called effector-triggered immunity (ETI). In the present study, the transcriptome analysis of the snow mould resistant genotype showed that the PTI pathway was activated (Fig. 7), particularly by the up-regulation of the expression level of calcium-dependent protein kinase *CDPK*, respiratory burst oxidase homolog *Rboh* and calcium-binding protein CaM/CML. Therefore, the activation of the PTI inhibits the snow mould pathogen from colonizing the plant tissues by increasing the production of reactive oxygen species and cell wall reinforcement. These results are similar to studies in *Festulolium*, where the resistant genotypes are characterized by high peroxidase activity, intensive lignification, callus formation and high concentrations of reactive oxygen species during the stage of early infection (within 6 days from inoculation)^31^.

Transcription factors, such as WRKY, play important roles in defense responses towards several plant pathogens^35^. The transcription level of WRKY genes are up-regulated by several stress factors, in particular pathogen infection^36^. In Arabidopsis, 49 out of 72 WRKY genes tested responded to bacterial infection or salicylic acid^37^, and 8 Arabidopsis WRKY genes (*WRKY 18*, *WRKY 38*, *WRKY 53*, *WRKY 54*, *WRKY 58*, *WRKY 59*, *WRKY 66*, and *WRKY 70*) were characterized as direct targets of NPR1, a key regulator of SA signalling^38^. In the present study, the resistant genotype showed high transcription levels of several WRKY genes such as *WRKY 70* and *WRKY 75*. Therefore, it is expected that the up-regulation of these genes will lead to the activation of the salicylic acid pathway^37^. Furthermore, our results also showed down-regulation of *WRKY 18* and *WRKY 33*, which are responsible for the activation of the JA pathway and the deactivation of the SA pathway^36^,^39^. In a study by Gaudet *et al*.^25^, the expression levels of *WRKY 34* and *WRKY 16* were up-regulated in snow mould resistant genotypes of winter wheat, which led to the activation of the JA pathway. Other studies showed that *M. nivale* infection is usually influenced by the physical and the chemical conditions of the plant tissue, thus the fungus behaves as a biotroph when the plant defense system is induced and the SA pathway is activated^9^,^10^.

The cross talk between cell morphogenesis and plant-pathogen interactions plays a crucial role in disease development^40^. Plants have developed a system for sensing pathogens and monitoring the cell wall integrity, upon which they activate defense responses that lead to a dynamic cell wall remodelling required to prevent disease^40^. Genes responsible for actin nucleation, aryl-alcohol dehydrogenase (NADP+) activity and cell differentiation were significantly enriched in the R genotype based on GO enrichment analysis by Fisher’s exact test (Fig. 6, Supplementary Table S4). Under GO term actin nucleation, we found that genes encoding actin-related protein 2 (*ARP2*), importin-β and serine threonine-protein kinase (*TOR*) were over-represented in the R genotype during infection. ARP2 in complex with ARP3 plays a central role in actin cytoskeletal formation^41^, and genetic experiments have indicated a role for this complex in the early stages of low temperature signalling^42^ and in the response to salt stress^43^. The importin- β subunit belongs to nuclear import receptors which play an essential roles in transferring defense proteins, such as nucleotide-binding and leucine-rich repeats (NB-LRRs), from the cytoplasm to the nucleus^44^, where they initiate defense signalling^45^. Moreover, protein kinases such as serine threonine-protein kinase (TOR) play a key role in signalling during pathogen recognition and subsequent activation of plant defense mechanisms^46^.

Under GO term aryl-alcohol dehydrogenase activity, a gene encoding a voltage-gated potassium channel beta subunit like was over-represented in the R genotype. Potassium (K^+^) plays many important regulatory roles in plant development and stress responses^47^. High K^+^ status decreases the occurrence of many diseases^48^. Furthermore, K^+^ affects plant hormonal pathways, i.e. the salicylic acid (SA) and jasmonic acid (JA) pathways^48^, which are involved in hypersensitive responses or acquired systemic resistance to pathogens. Recent studies^49^ showed that overexpression of *GmAKT2* encoding a K+ transporter significantly increased K+ concentrations and consequently resistance to soybean mosaic virus in transgenic soybean^49^.

Under cell differentiation gene ontology, we detected that the genes encoding glycerol-3-phospahate-1 (G3P) transporter and prefoldin (PFD) were over-represented in the snow mold infected R genotype. The G3P transporter is an important component of carbohydrate and lipid metabolic processes. G3P levels in *A. thaliana* plants were previously associated with defense to the hemibiotrophic fungal pathogen *Colletotrichum higginsianum*^50^. Infection of *A. thaliana* with *C. higginsianum* showed an increase in G3P levels and a concomitant enhanced resistance in the host^50^. Prefoldin proteins are required for the cytoplasmic folding of actin and tubulin monomers during cytoskeleton assembly^51^. Recent studies^52^ showed that prefoldin 6 interacts with two *P. syringae* effectors and defense regulatory protein EDS1 (enhanced disease susceptibility 1). Additionally, prefoldins 3 and 5 have been shown to play essential roles in tolerance to salt stress in Arabidopsis^53^. Significant enrichment of these GO terms in the R genotype show that these gene systems are involved in defense responses to pink snow mould infection in perennial ryegrass.

## Conclusions

In this study, a susceptible (S) and a resistant (R) genotype of *L. perenne* cv. Fagerlin were found to be significantly different in resistance, as measured by relative regrowth, and accumulation of *M. nivale* DNA, as quantified by real-time qPCR. RNA sequencing of transcriptome responses of non-cold acclimated plants to early infection by an aggressive *M. nivale* isolate identified differentially expressed genes between the S and R genotype. Many pathogen related genes were found to be upregulated during snow mould infection in the resistant genotype. Further GO enrichment analysis confirmed that specific GO terms related to plant defense are over-represented in the resistant genotype. The list of putative candidate defense associated genes and cell morphogenesis associated genes identified in this study might provide a scientific basis for further investigations to obtain more in-depth understanding of host-pathogen interactions and development of resistant cultivars by marker-assisted breeding.

## Materials and Methods

### Plant materials and growth conditions

For screening of snow mould resistance and quantification of fungal DNA, mother plants of eight randomly selected genotypes (A-F, M and K) of perennial ryegrass, cultivar Fagerlin, were divided into multiple ramets and planted in a fertilized soil mixture (Gartnerjord, TJERBO) and grown in the greenhouse at 20–22 °C (day/night) and 18 hours light period with light intensity (CONSTANTCOLOR CMH lamps 400W) at about 200–250 μmol m^−2^ s^−1^. The plants were fertilized weekly with a mixture of 80 g/L KRISTALON fertilizer 9-5-25 (N-P-K) and 60 g/L of YARALIVA CALCINIT 15.5-0-0 (Yara International ASA, Oslo, Norway), diluted to a conductivity of 2 mS/cm. For the transcriptome studies, plants from the eight genotypes were randomly selected and placed on four trolleys (100 × 60 cm) and moved to controlled growth chamber at 18/20 °C (day/night) temperature, with light intensity of 220–240 μmol m^−2^ s^−1^ for four weeks. Perennial ryegrass is often infected with endophyte *Epichloë festucae* var. *lolii*. However, the cultivar Fagerlin used in this study, is not infected with endophyte *Epichloë festucae* var. *lolii* (pers. communication with the forage grass breeder Petter Marum, Graminor AS, the owner of the variety). Further to be sure, we performed PCR to screen the eight genotypes of Fagerlin for endophytes with the β-tubulin (*TUB2*)^54^, translation elongation factor 1-a (*TefA*)^55^ and the soft (*SO*)^55^ genes (which are essential in screening for endophyte infection). We did not detect any traces of these genes in our genotypes, indicating that Fagerlin is free of endophyte infection.

### Snow mould resistance test

Isolate 200231 of *M. nivale* (isolated from *L. perenne* at Ås, Norway (59°N) was obtained from the fungal culture collection at the Norwegian Institute of Bioeconomy Research (NIBIO), Ås, Norway. The inoculum was prepared according to Tronsmo^56^ and Hofgaard *et al*.^57^. Briefly, the fungus was incubated at 9 °C, in darkness, on potato dextrose agar (PDA) for two weeks. An Erlenmeyer flask containing 100 ml potato dextrose broth (PDB) was then inoculated with four plugs (5mm diameter) of fungal mycelium and incubated at 15 °C in darkness. Fungal mycelium was harvested after 10 days by filtering through cheesecloth. The mycelium was homogenized in distilled water containing 0.01% TWEEN 20 (SIGMA) using an ULTRA TURRAX. The inoculum was diluted to an optical destiny of 0.5 at 430 nm. Plants were inoculated by spraying (1 ml inoculum per plant on average) and the control plants were sprayed with distilled water. After inoculation, the plants were incubated under artificial snow cover by covering the plants with moist cellulose tissue paper and black plastic sheets, then placed in a cold chamber at 2 °C in darkness for six and eight weeks. Each week during incubation, the plants were repositioned in the room.

After incubation, the plants were moved to a greenhouse at 20–22 °C and 18 hours of light for recovery. The plants were cut at five cm above the soil surface and allowed to regrow for two weeks. The regrown plants were harvested (all parts above soil surface) and dried at 60 °C for three days in order to measure dry weight (g DW plant^−1^). Relative regrowth was calculated for each inoculated plant as the dry weight divided by the average dry weight of non-inoculated plants within the same genotype. Relative regrowth values approaching 1 represents resistant plants. Disease severity was visually scored two days later according to the following scale: 0 = no green tillers, 1 = some green tillers visible, 2 = green tillers found in less than half of the total plant area, 3 = green tillers found in more than half of the plant area, and 4 = green tillers observed in the whole plant area. After visual assessment of the symptoms, plant leaves and stems were harvested (5 cm above soil surface) and kept at −20 °C for DNA extraction for fungal quantification.

### Real-time PCR for fungal quantification

Plant materials (leaves and stems above five cm from soil) were collected from the eight genotypes at two different time points (6 and 8 weeks after inoculation), from the genotypes F and M also at 1 and 4 days after inoculation. For the samples collected 6 and 8 week after inoculation, plant materials were stored at −20 °C until DNA was extracted. For samples collected after 1 and 4 days, the same plant materials used for gene expression analysis (see section below) were utilized for fungal DNA quantification. For DNA extractions, samples were frozen quickly in liquid N_2_ and ground using mortar and pestle. DNA was extracted from the ground plant tissue using DNeasy Plant Mini Kit (QIAGEN) according to manufacturer’s protocol (Qiagen Inc., Germany). The quality of the extracts was measured using a NANODROP ND-1000 UV–Vis Spectrophotometer (NanoDrop Technologies, Wilmington, DE, USA) and visualized by electrophoresis through 1.5% agarose gels.

Real-time PCR primers specific for *M. nivale* were designed based on the *EF-1a* gene sequence by Glynn *et al*.^15^ using PRIMER EXPRESS software version 2 (Applied Biosystems, Foster City, USA) based on the following parameters; amplicon Length of 50 to 150 bases (for optimum PCR efficiency), primer length of 20 bases, melting temperature (Tm) of 58 °C to 60 °C (Optimal 59 °C), G + C content being between 30 and 80%; and the last five nucleotides at the 3′ end do not contain more than two G + C residues. The following primer set was chosen based on the regions of identity within the *EF-1a* gene sequence between isolates, forward primer EF1-F: 5´-GGTCTTGGCTTGCACAAACA-3´ and reverse primer EF1-R: 5´- AGCACAACAGGCGTGGATAAG -3´. Quantification of plant and fungal DNA was carried out by real time PCR in a total volume of 25 μl, using 2x SYBR Green PCR master mix (Applied Biosystems), 300 nM of each primers (Invitrogen Ltd, UK) and 2 μl of 10x diluted template DNA. Specific real time PCR primers for the plant housekeeping gene *LpGAPDH*^58^ were used as internal control for plant DNA. PCR was performed on Applied Biosystems 7900HT instrument with a standard 96-well block (Applied Biosystems). For all the PCR reactions the following cycling parameters were used: 50 °C for 2 min, 95 °C for 10 min, 40 cycles of 95 °C for 15 s and 60 °C for 1 min followed by dissociation curve analysis at 60 °C–95 °C. The data was analysed using SEQUENCE DETECTION SOFTWARE (SDS) Version 2.2.1 (Applied Biosystems). The amount of fungal and plant DNA in the samples were quantified by a standard curve algorithm based on cycle threshold value (Ct) using a 10-fold dilution series of known amount of DNA, starting with 5 ng for fungal DNA and 100 ng for plant DNA and three technical replicates. Samples were tested in two technical replicates. The amount of fungal DNA was calculated as pg fungal DNA per μg plant DNA for each sample.

### Tissue sampling and RNA extraction for gene expression analysis

Leaf samples were collected from genotype F, a susceptible genotype (hereafter termed S) and genotype M, a resistance genotype (actually less susceptible, here after termed R), which had been exposed to three different treatments: 1: Non-inoculated and non-incubated plants (NI-NI) (Control plants kept in ambient temperature), 2: Non-inoculated plants that were incubated under artificial snow-cover for four days (I-NI), and 3: Inoculated plants that were incubated under artificial snow-cover for four days (I-I), with two biological replicates, a total number of 12 samples. The collected samples were immediately placed in liquid nitrogen and stored at −80 °C until used for RNA extraction. The frozen leaf samples were crushed with a pestle and mortar and total RNA was extracted using the PURE LINK RNA MINI KIT (Life technologies, USA) and PLANT RNA ISOLATION AID (Life technologies, USA). On-Column DNAse kit was used to remove the DNA contamination. The concentration and quality was checked using the NANODROP (Nanodrop Technologies, Wilmington, DE, USA) and BIOANALYZER (Agilent Technologies, Palo Alto, CA, USA) equipment.

### cDNA library construction and Illumina sequencing

Twelve RNA samples with RIN (RNA Integrity Number) values above seven were used to construct separate cDNA libraries with fragment lengths of 200 bp (±25 bp). Then, paired-end sequencing was performed by GATC Biotech Ltd., Germany (http://www.gatc-biotech.com/en/index.html) using the Illumina sequencing platform (HISEQ 2000). Real time analysis (RTA) output was analysed using the CASAVA software (version 1.6, Illumina), generating pass filtering FastQ files with Qphred +64 quality values. Paired-end reads with a length of 100 bp were generated. The reads were deposited in the EMBL-EBI ArrayExpress Archive, under accession number E-MTAB-4459. The quality of the reads was analysed using FastQC (http://www.bioinformatics.babraham.ac.uk/projects/fastqc/).

### *De novo* and reference based transcriptome analysis

After trimming adapter sequences and filtering low quality reads using the sickle program (https://github.com/najoshi/sickle/blob/master/README.md), the bioinformatics pipeline (Supplementary Fig. S4) was followed for *de novo* assembly and further detection of differentially expressed genes. Briefly, the clean reads derived from the two genotypes susceptible (S) and resistant (R) were used to construct separate *de novo* assemblies for each genotype using the Trinity assembler (release 2013-02-25)^59^ with the following settings; Trinity.pl --seqType fq --JM 20G --left F_1_Lolium.fq-QT.gz --right F_2_Lolium.fq-QT.gz --CPU 16 -min_contig_length 200 --SS_lib_type FR --full_cleanup --min_kmer_cov 2 --output Trinity_201 2 > &1 > logfile.lolium-F. The *de novo* assembled transcripts were then used as a reference to map back the individual reads by Bowtie. Further, we estimated transcript abundances in each genotype and treatment combination using RSEM version 1.1.11^60^. A maximum of one mismatch (–bowtie-n 1) was allowed in the seed region of the reads. In another approach, to facilitate comparison of the two genotypes, we aligned all clean reads from each genotype and treatment combination to a reference transcriptome of an inbred *L. perenne* genotype, generated from a combination of root, stem, leaf sheath, leaf and meristem samples^22^, and estimated transcript abundance as described above.

### *De novo* assembly validation by CEGMA

CEGMA software (version 2.4)^21^ was used to assess the completeness of the S and R transcriptome assembly datasets. This program assesses the presence and coverage of a set of 248 extremely conserved core eukaryotic genes (CEGs). It is routinely used for evaluating genomic assemblies, however, it has also been used for evaluating transcriptome assemblies^61^,^62^. The software was run with default parameters with the included reference dataset of 248 ultra-conserved Core Eukaryotic Genes (CEGs).

### Identification of differentially expressed genes, BLAST and functional annotation

Gene expression levels were measured as expected number of fragments per kilobase of transcript sequence per millions mapped reads (FPKM)^63^. The transcript matrix files derived from RSEM program were processed with the edgeR program^64^ using perl script (DE_analysis.pl) in the Trinity pipeline for detecting differentially expressed genes determined with a False Discovery Rate (FDR) of 0.05. Briefly, pairwise comparisons were carried out between all the selected time points and edgeR^64^ analysis was performed by fitting normalized count data with a generalized linear model (GLM) estimating a negative binomial distribution to the calculated mean values of the two biologically independent samples. For each gene, fold changes and P values (pval) as well as P values adjusted (padj) for multiple testing with the Benjamini-Hochberg procedure^65^, were used to control FDR. The sequence with padj of less than 0.05 was deemed to be significantly differentially expressed genes (DEGs). The variance stabilized data obtained from edgeR^64^ was used as input for clustering, and for constructing multidimensional scaling plots using R integrated in the Trinity pipeline. The transcripts showing differential expression at any time point during snow mould infection were clustered using a K-means clustering algorithm. VennPlex program^66^ was used to generate Venn diagrams showing up-, down- and contra-regulated transripts.

The DEGs were annotated using Blast2GO^23^. An E-value threshold of 10^e-06^ was used for the BLASTx search, and 10^e-10^ for the annotation, with a cut-off value of 55 and a GO weight Hsp-hit value of 20. The enrichment analysis for the differential gene ontology term distribution was performed with a p-value significance cut-off value of 0.01. Gene ontology classifications of differentially expressed genes in the resistant (R) and susceptible (S) genotypes were generated using the web histogram tool WEGO^24^. Pathway analysis was performed using the KEGG function implemented in the Blast2GO^23^ tool.

### Validation of RNAseq expression profiles by qRT-PCR

Expression patterns of five defense related genes (*chitinase 2*, *chitinase 5*, *WRKY*, *PR3*, *PR1* and *PR5*) differentially expressed between I-NI and I-I samples identified in this transcriptome studies were analysed using qRT-PCR. The same RNA used for sequencing was used for validating the genes by qRT-PCR. Based on the transcriptome sequences of the five genes, primers (Supplementary Table S3) were designed using primer express software version 2 (Applied Biosystems, Foster City, USA). Efficiency test of the primers was performed on different samples for normalization of the expression level. The EXPRESS two-Step qRT-PCR kit, which includes the SuperScript VILO cDNA Synthesis kit, was used for generating the single-stranded cDNA that was later used for quantifying the amount of specific gene expression using forward and reverse primers, following the manufacturer’s instructions. cDNA synthesis was done using up to 2.5 μg of the total RNA in 20 μl reaction. Five μl of cDNA (5x diluted) was used in each well of Fast Optical 96 well plate along with other components making the total volume 20 μl. The fast cycling program was then set at 95 °C for 20 sec, 40 cycles of 95 °C for three sec (denaturation) and 60 °C for 35 sec (annealing). Then each plate (with samples) for each gene with a bar code was placed in ABI7500 qRT-PCR machine. The SYBR Green dye was used to detect the amplified products. The expressions of the specific genes were normalized by using *LpGAPDH* (EC 1.2.1.12) as the reference gene. The expression of the target gene relative to the reference gene at 4 days after inoculation using the 2^−ΔΔCT^ method where the ΔΔCT = (CT of target – CT of reference) 4 days after inoculation – (CT of target – CT of reference) before inoculation, which gives the mean relative expression of target genes at this time point^67^.

## Additional Information

**Accession codes:** The raw data generated in this study were deposited in the EMBL-EBI ArrayExpress Archive, under accession number (E-MTAB-4459). Snow mould resistant (R) and susceptible (S) *de novo* transcriptome assemblies generated by trinity program are deposited in DRYAD Digital Repository along with the sequences (ESTs) of candidate genes detected for snow mould resistance and sequences for the genes under GO terms significantly over represented in resistant genotype in this study (http://datadryad.org/resource/doi:10.5061/dryad.mc7c1).

**How to cite this article**: Kovi, M. R. *et al*. Global transcriptome changes in perennial ryegrass during early infection by pink snow mould. *Sci. Rep.* **6**, 28702; doi: 10.1038/srep28702 (2016).

## Supplementary Material

Supplementary Information

Supplementary Table S4

Supplementary Table S5

## Figures and Tables

**Table 1:** Characteristics of the *de novo* transcriptome assemblies.

	Susceptible genotype (S)	Resistant genotype (R)
Min. contig length (bp)	201	201
N50 (bp)	1,784	1,672
Max. contig length (bp)	17,632	12,882
Total no. of contigs	261,978	188,355
Sum of the reads	178,000,000	165,000,000

**Table 2:** Results of CEGMA analysis for *de-novo* assembly validation.

Out of 248 CEGs^1^	Resistant genotype (R)	Susceptible genotype (S)
% of fully represented	82.66	93.95
% of at least partially represented	90.73	98.79
Average number of orthologs per CEG	3.72	3.83
% of detected CEGs with more than 1 ortholog	96.10	97.00

^1^CEGs: Core Eukaryotic Genes

**Table 3:** List of differentially expressed genes that can be considered as potential candidate genes involved in response to *M. nivale* in two Lolium perenne, cv. Fagerlin genotypes, R (resistant genotype), and S (susceptible genotype).

Sequence ID	*Arabidopsis thaliana*homologue	*Brachypodium distachyon* homologue	Description	Log^a^ FC I-NI (S/R)	Log^b^ FC I-I (S/R)
comp10786_c0_seq2		Bradi1g36400.2	26s protease	8.78	−11.8
comp11656_c0_seq1	AT3G08550.1	Bradi2g07890.3	Abscisic acid insensitive protein	−2.94	11.58
comp11730_c0_seq1	AT4G16830.3	Bradi5g10027.1	Abscisic acid protein	10.98	−0.36
comp12003_c0_seq2	AT4G16830.3	Bradi5g10027.1	Abscisic acid protein	−11.65	−1.36
comp13445_c0_seq1	AT2G27730.1	Bradi4g38600.2	Atpase inhibitor protein	10.06	−11.4
comp13446_c0_seq1	AT5G62000.4	Bradi2g59480.1	Auxin response factor 2	−0.05	11.57
comp14875_c0_seq1	AT5G62000.4	Bradi2g59480.1	Auxin response factor 2	11.15	11.57
comp15074_c0_seq2	AT1G56220.4	Bradi4g31110.2	Auxin-repressed protein	−4.60	11.96
comp15317_c0_seq1	AT2G41140.1	Bradi1g61637.1	Calcium -dependent protein kinase 1	−0.51	7.07
comp15765_c0_seq1	AT5G23580.1	Bradi4g24390.1	Calcium-dependent protein kinase sk5	1.11	10.83
comp16692_c0_seq1	AT1G35670.1	Bradi4g24390.1	Calcium-dependent protein kinase sk5	8.36	9.74
comp17044_c0_seq1	AT5G57580.1	Bradi3g05760.2	Calmodulin binding protein	0.37	−10.5
comp17054_c0_seq1	AT3G49050.1	Bradi2g00831.1	Calmodulin-binding heat shock protein	0.48	−9.68
comp17565_c0_seq2	AT3G16920.1	Bradi4g34040.1	Chitinase 2	−9.07	9.47
comp22298_c0_seq1	AT3G54420.1	Bradi5g14430.1	Chitinase 5	3.42	3.16
comp23317_c0_seq2	AT3G12500.1	Bradi3g32340.1	Class II chitinase	−0.71	3.26
comp23443_c0_seq2	AT2G02120.1	Bradi3g49380.1	Defensin precursor	−9.00	0.40
comp23963_c0_seq1	AT3G14470.1	Bradi1g29560.2	Disease resistance protein	1.59	11.71
comp24194_c0_seq2	AT1G64160.1	Bradi1g20185.1	Disease resistance protein	1.38	3.17
comp24580_c0_seq2	AT3G46730.1	Bradi1g51961.2	Disease resistance protein 3	7.97	3.57
comp24988_c1_seq1	AT1G72540.1	Bradi1g51961.2	Disease resistance protein 3	6.42	−2.26
comp25199_c0_seq1	AT3G14460.1	Bradi2g25327.1	Disease resistance protein rga3	5.05	−2.01
comp26954_c0_seq8	AT3G46730.1	Bradi3g15593.1	Disease resistance protein rpm1	9.76	8.47
comp27012_c0_seq1	AT3G46730.1	Bradi4g24887.1	Disease resistance protein rpm1	1.10	9.53
comp27236_c0_seq2	AT1G59780.1	Bradi3g15593.2	Disease resistance protein rpm1	12.59	6.93
comp27390_c0_seq1	AT3G07040.1	Bradi4g35317.1	Disease resistance protein rpm1	9.81	1.00
comp27751_c0_seq17	AT3G07040.1	Bradi4g35317.1	Disease resistance protein rpm1	11.93	2.13
comp28444_c0_seq2	AT1G58602.1	Bradi4g21950.2	Disease resistance protein rpp13	−1.17	10.66
comp28535_c0_seq2	AT3G20770.1	Bradi1g63780.1	Ethylene signal transcription factor	9.53	2.16
comp28907_c0_seq3	AT2G27050.1	Bradi1g63780.1	Ethylene signal transcription factor	3.70	−11.2
comp28998_c0_seq2	AT5G03280.1	Bradi4g08380.1	Ethylene-insensitive protein 2-like	6.49	10.66
comp29029_c0_seq9	AT1G53910.3	Bradi1g46690.3	Ethylene-responsive transcription factor 1	0.25	2.03
comp29851_c0_seq7	AT3G14230.3	Bradi2g02100.1	Ethylene-responsive transcription factor crf4	−9.85	0.94
comp30083_c0_seq2	AT1G53910.3	Bradi1g46690.3	Ethylene-responsive transcription factor rap2	2.42	−0.74
comp30409_c0_seq3	AT1G55270.1	Bradi3g01360.3	F-box kelch-repeat protein	0.85	12.01
comp30635_c0_seq3		Bradi3g31520.1	F-box protein	2.16	−11.8
comp30748_c0_seq3	AT2G42620.1	Bradi1g49120.2	F-box protein ore9-like	0.54	9.56
comp30853_c0_seq1	AT2G24270.4	Bradi3g36930.1	Glyceraldehyde-3-phosphate dehydrogenase	12.60	−12.8
comp30959_c0_seq10	AT3G25530.1	Bradi3g46080.1	Glyoxylate reductase 1	4.57	11.89
comp31014_c0_seq1	AT5G02500.1	Bradi1g03720.1	Heat shock protein 70	5.10	13.11
comp31072_c0_seq5	AT5G02500.1	Bradi1g03720.1	Heat shock protein 70	4.02	12.15
comp31301_c0_seq19	AT5G63890.1	Bradi1g17340.1	Histidinol dehydrogenase	1.37	−12.7
comp31318_c0_seq3	AT4G14420.1	Bradi1g75100.2	HR-like lesion-inducing protein	4.10	−12.7
comp31337_c1_seq2	AT1G15690.2	Bradi1g30550.1	Inorganic H pyrophosphatase protein	−4.93	13.84
comp31380_c0_seq1	AT2G38540.1	Bradi4g25750.1	Lipid transfer protein	−1.76	3.91
comp31450_c0_seq51	AT2G38540.1	Bradi4g25750.1	Lipid transfer protein	−2.82	1.86
comp31553_c0_seq3	AT2G42880.1	Bradi2g45870.1	MAP kinase protein	12.53	−0.05
comp31649_c0_seq29	AT3G55270.1	Bradi2g37450.2	MAP kinase phosphatase	13.85	−0.09
comp31684_c0_seq5	AT5G56580.1	Bradi1g75150.1	MAP kinase protein	12.57	−3.52
comp31934_c0_seq20	AT1G53570.5	Bradi3g45790.1	MAPkkk protein kinase	0.47	0.57
comp31966_c0_seq4	AT1G53570.2	Bradi5g10670.2	MAPkkk protein kinase	10.90	0.28
comp31978_c0_seq6	AT1G07180.1	Bradi2g53970.1	NAD(P)H dehydrogenase 1	0.06	12.56
comp32014_c0_seq2	AT3G14470.1	Bradi1g29560.2	NB-ARC disease resistance protein	2.33	-7.97
comp32363_c0_seq37	AT4G26090.1	Bradi5g15560.1	NB-ARC disease resistance protein	8.92	1.35
comp33360_c0_seq1	AT2G26040.1	Bradi1g64920.1	Pathogenesis-related protein 1	−1.44	12.21
comp33451_c0_seq1	AT4G25780.1	Bradi1g57540.1	Pathogenesis-related protein 1	1.75	4.48
comp33568_c0_seq1	AT3G04720.1	Bradi4g14930.1	Pathogenesis-related protein 4	−3.31	1.58
comp34254_c0_seq1	AT1G75050.1	Bradi4g05440.1	Pathogenesis-related protein 5	0.50	5.60
comp34926_c0_seq1	AT1G78780.2	Bradi2g08707.1	Pathogen-related protein	−8.18	0.37
comp35213_c0_seq1	AT1G09570.2	Bradi1g10520.2	Phytochrome A	−5.79	−10.4
comp35885_c0_seq1	AT4G35470.1	Bradi3g33990.1	Plant intracellular ras group-related LRR 4	9.20	12.03
comp35990_c0_seq1	AT1G64060.1	Bradi2g19090.5	Respiratory burst oxidase protein 2	−0.48	9.35
comp36198_c0_seq1	AT2G39840.1	Bradi3g55614.3	Serine threonine protein phosphatase pp1	3.01	−12.06
comp36434_c0_seq1	AT2G39840.1	Bradi3g55614.3	Serine threonine protein phosphatase pp1	3.03	−11.4
comp37190_c0_seq1	AT4G33950.1	Bradi1g07620.1	Serine threonine-protein kinase	1.24	−0.32
comp38150_c0_seq1	AT3G13380.1	Bradi4g27440.1	Serine threonine-protein kinase	−0.28	0.37
comp41496_c0_seq1	AT4G33080.1	Bradi2g33530.2	Serine threonine-protein kinase cbk1	−1.22	−8.39
comp42382_c0_seq1	AT4G33080.2	Bradi2g33530.1	Serine threonine-protein kinase cbk1	−1.84	−8.13
comp43183_c0_seq1	AT5G02800.1	Bradi1g76362.2	Serine threonine-protein kinase pbs1	0.06	−9.37
comp44101_c0_seq1	AT5G22840.1	Bradi1g08660.2	Serine threonine-protein kinase srpk2	10.60	−1.25
comp45052_c0_seq1	AT2G13360.1	Bradi3g39750.2	Serine-glyoxylate aminotransferase	−0.71	1.41
comp46601_c0_seq1	AT3G15610.1	Bradi1g36840.1	Serine-threonine kinase receptor-associated	1.95	−4.15
comp48325_c0_seq1	AT2G45950.1	Bradi1g62007.1	SKP1-like protein 21	−1.25	8.57
comp49870_c0_seq1	AT4G11650.1	Bradi4g05440.1	Thaumatin domain family protein	−9.07	12.16
comp5056_c0_seq1	AT4G11650.1	Bradi3g07960.1	Thaumatin pathogenesis-related protein 3	−9.96	3.14
comp64771_c0_seq1	AT2G02760.1	Bradi2g05400.2	Ubiquiting-conjugating enzyme 2	−1.97	13.67
comp6936_c0_seq1	AT4G31800.2	Bradi1g30870.1	WRKY DNA-binding protein 18	−9.91	−2.84
comp73317_c0_seq1	AT4G31800.2	Bradi3g06070.1	WRKY DNA-binding protein 18	1.95	−1.39
comp7627_c0_seq1	AT5G56270.1	Bradi4g33370.1	WRKY DNA-binding protein 2	−10.43	10.95
comp76423_c0_seq1	AT2G38470.1	Bradi2g00280.1	WRKY DNA-binding protein 33	2.32	−5.45
comp7959_c0_seq1	AT5G64810.1	Bradi2g18530.1	WRKY DNA-binding protein 51	4.05	0.34
comp8111_c0_seq1	AT1G29280.1	Bradi2g49906.1	WRKY DNA-binding protein 65	−8.24	0.05
comp8129_c0_seq1	AT2G46400.1	Bradi1g17660.1	WRKY DNA-binding protein 70	−0.47	1.84
comp8631_c0_seq2	AT5G13080.1	Bradi4g19060.1	WRKY DNA-binding protein 75	0.53	1.49
comp8708_c0_seq1	AT3G55980.1	Bradi4g05990.2	Zinc finger protein 33	−0.97	9.28
comp8930_c0_seq1	AT2G16485.1	Bradi4g35977.2	Zinc finger protein 44	−2.49	0.13
comp9122_c0_seq1	AT2G27580.1	Bradi1g06036.1	Zinc finger stress-associated protein 6	0.39	9.21
comp9340_c0_seq1	AT1G07360.1	Bradi1g48140.1	Zinc finger protein 40	0.20	−9.68
comp9759_c0_seq1	AT3G12630.1	Bradi3g39850.1	Zinc finger stress-associated protein 5	−10.85	−9.95

^a^The log_2_ of the fold change between the resistant (R) and susceptible (S) genotype under non-inoculated and incubated conditions after 4 days of incubation (I-NI).

^b^The log_2_ of the fold change between the resistant (R) and susceptible (S) genotype under inoculated and incubated (I-I) conditions after 4 days of incubation.
