# Supplementary material for: Global transcriptome changes in perennial ryegrass during early infection by pink snow mould
Source: Sci Rep. 2016 Jun 27;6:28702. doi: 10.1038/srep28702 (PMC4921834; doi:10.1038/srep28702)
Supplement: Supplementary Information [file srep28702-s1.doc]

# Global transcriptome changes in perennial ryegrass during early infection by pink snow mould

Mallikarjuna Rao Kovi^1^, Mohamed Abdelhalim^1^, Anil Kunapareddy^1^, Åshild Ergon^1^, Anne Marte Tronsmo^1^, May Bente Brurberg^1,2^, Ingerd Skow Hofgaard^2^, Torben Asp^3^ and Odd Arne Rognli^1§^

*^1^Department of Plant Sciences, Norwegian University of Life Sciences, NO-1432 Ås, Norway*

*^2^Division of Biotechnology and Plant Health, Norwegian Institute of Bioeconomy Research (NIBIO), NO-1432 Ås, Norway*

*^3^Department of Molecular Biology and Genetics, Aarhus University, Slagelse, Denmark*

^§^Corresponding author: [odd-arne.rognli@nmbu.no](mailto:odd-arne.rognli@nmbu.no)


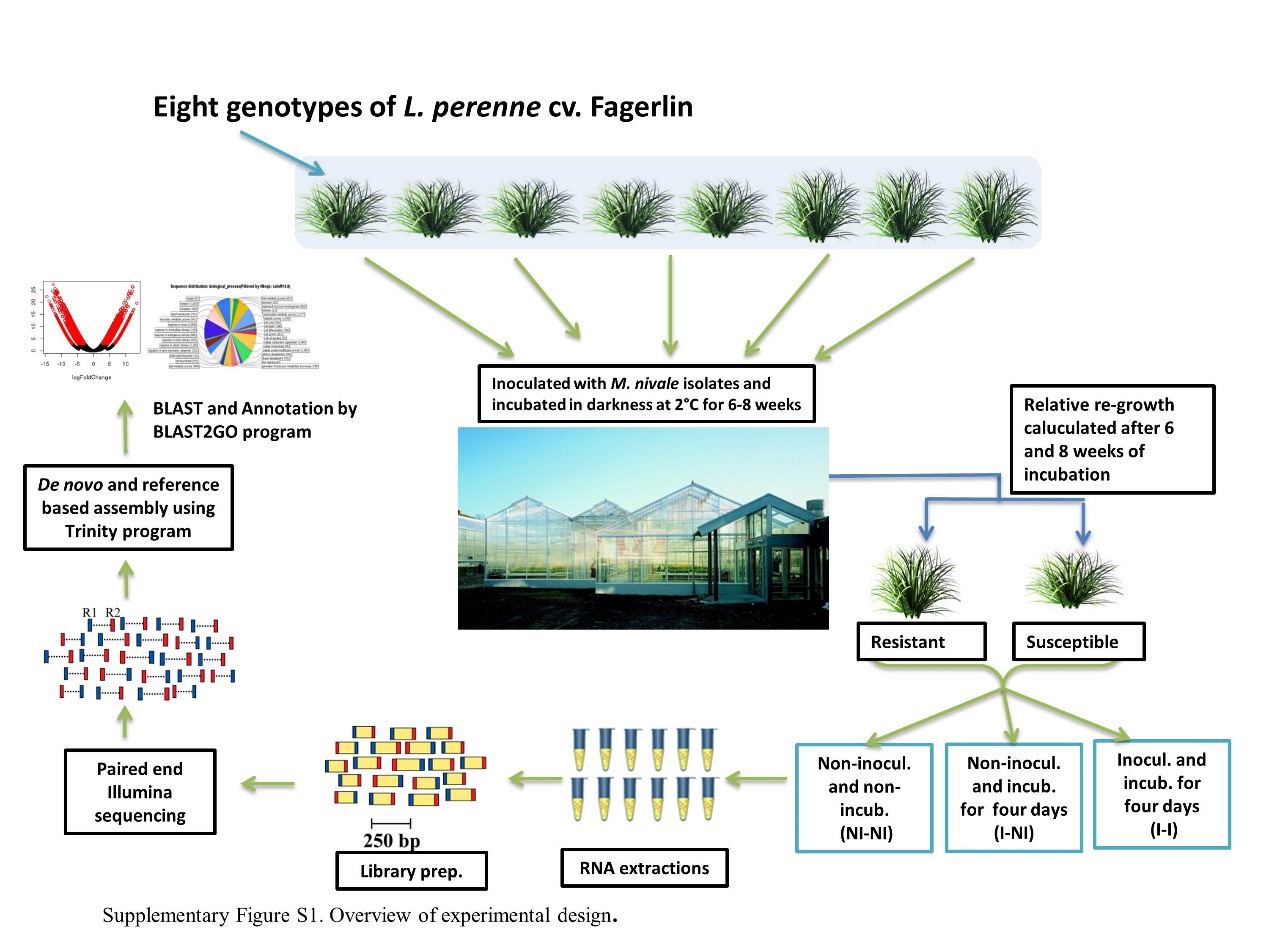


Supplementary Figure S1. Outline of experimental design.


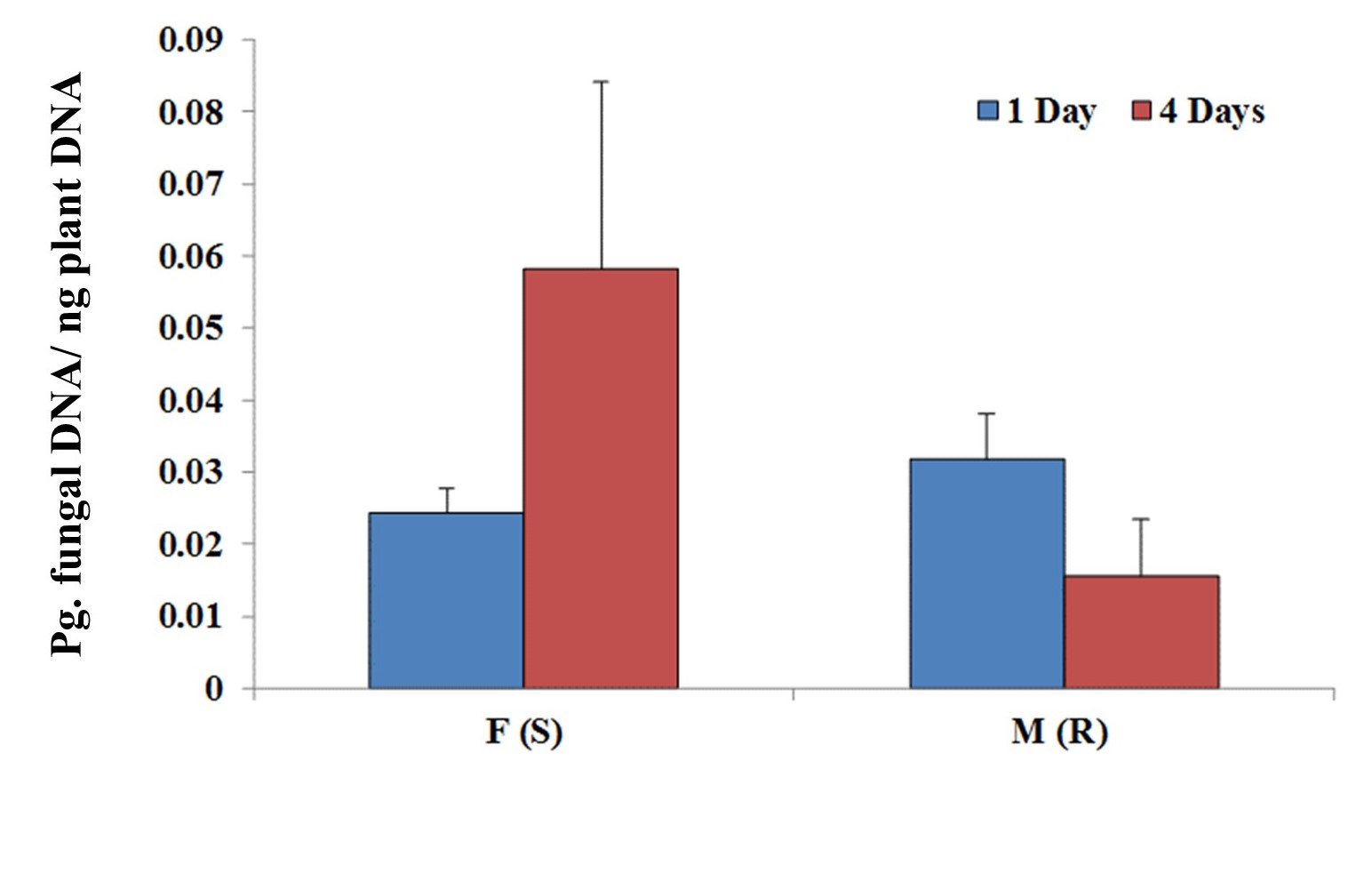


Supplementary Figure S2. Amount of fungal DNA (pg. fungal DNA/ng plant DNA) in two genotypes of *L. perenne* cv. Fagerlin (resistant genotype M(R) and susceptible genotype F(S)) inoculated with *M. nivale* (isolate 200231). Samples were collected 1 and 4 days after inoculation. Error bars indicate standard errors of the mean.


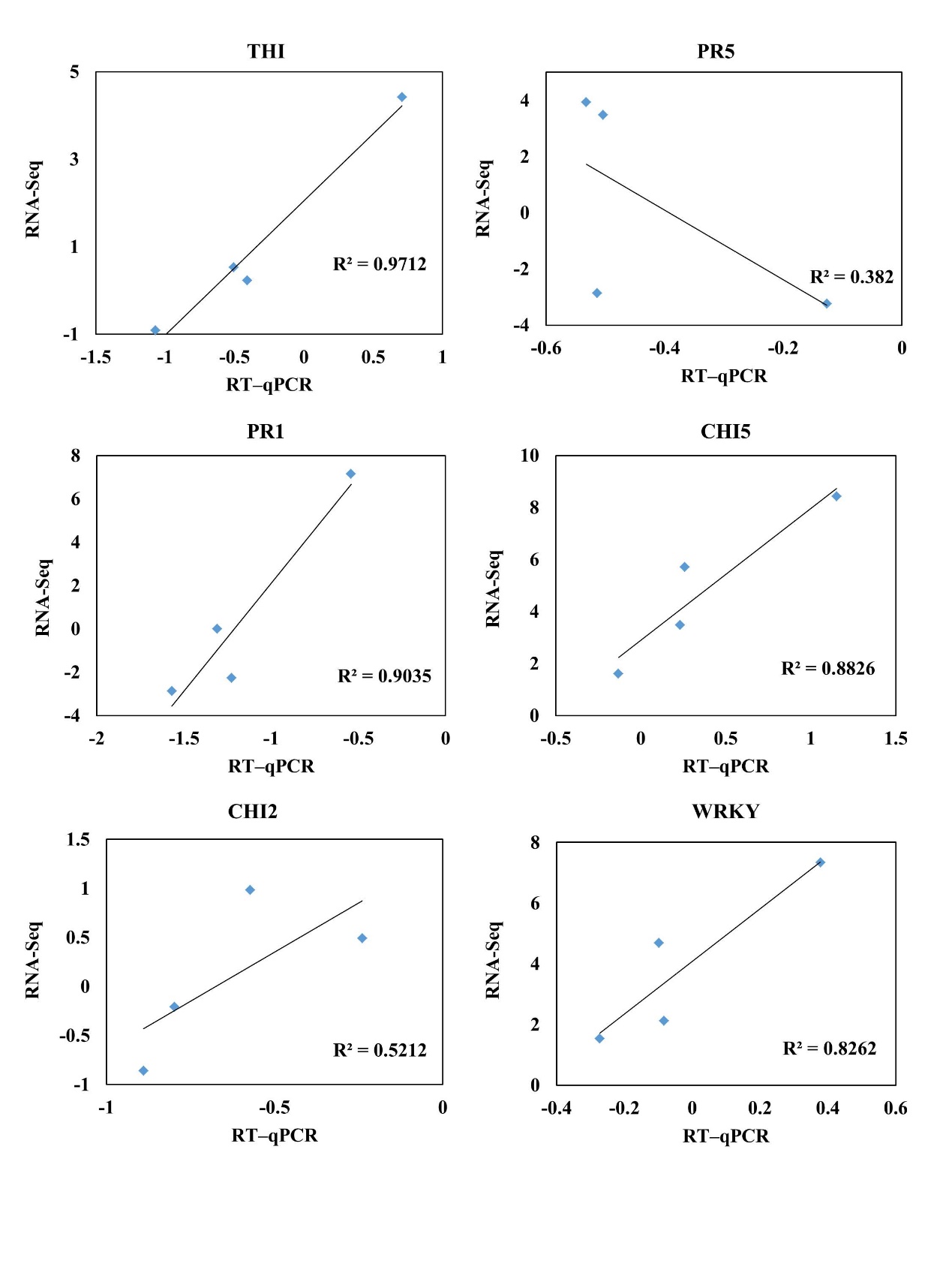
Supplementary Figure S3. Validation of gene expression of six selected genes by qRT-PCR. CHI2; Chitinase 2, CHI5; Chitinase 5, WRKY; WRKY transcription factor, THI; Thaumatin-like PR3, PR1 ; pathogenesis-related protein 1, PR5; pathogenesis-related protein 5. Scatter plots shows the correlation between RNA-Seq and qRT-PCR fold change.


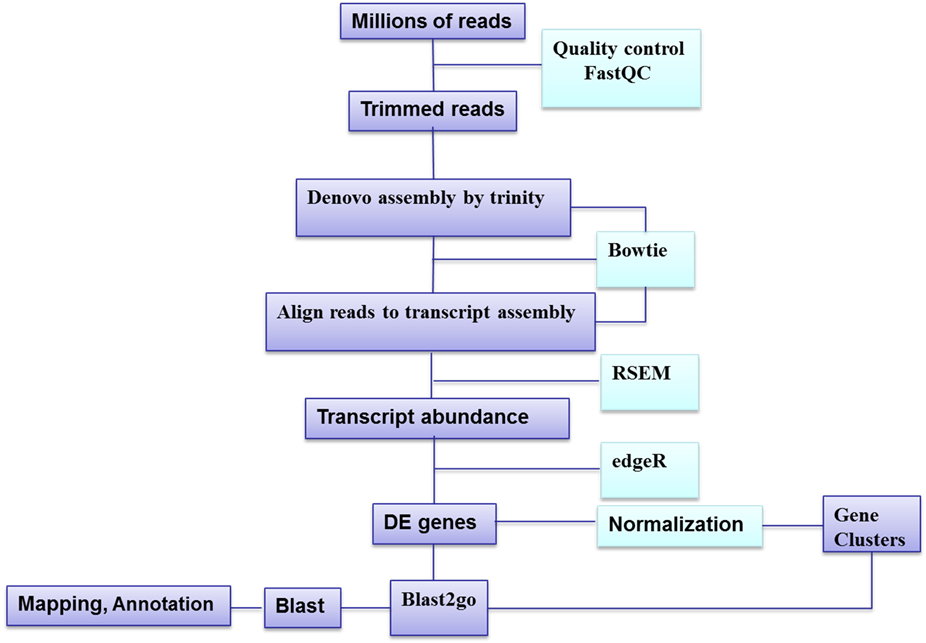


Supplementary Figure S4. Work flow of RNA sequence data analysis.

Supplementary Table S1. Correlations between relative regrowth (g dry weight), visual scoring of symptoms and the amount of fungal DNA in plant tissue of 8 genotypes of *L. perenne* cv. Fagerlin, after inoculation with *M. nivale* and incubation for 6 and 8 weeks at +2 °C.

| Traits | Relative regrowth^a^ | Visual scoring of symptoms^b^ |
| --- | --- | --- |
| Visual scoring of symptoms | 0.06 ^ns^ |  |
| The amount of fungal DNA^c^ | 0.0 ^ns^ | 0.489 ^*^ |

^*^ P≤ 0.05; ^ns^ non-significant. ^a^ Relative regrowth (dry weight of inoculated plants divided by dry weight of non-inoculated plants) after incubation under artificial snow cover followed by two weeks of regrowth. ^b^Visual assessment was done according to the following scale: 4 = no green tillers, 3 = some green tillers visible, 2 = green tillers found in less than half of the total plant area, 1 = green tillers found in more than half of the plant area, and 0 = green tillers observed in the whole plant area. ^c^ Amount of fungal DNA (pg. fungal DNA/ng plant DNA).

Supplementary Table S2. List of pathway-enriched differentially expressed genes in the resistant (R) and susceptible (S) genotype under incubated and inoculated (I-I) conditions after 4 days of incubation.

| Pathway | Differentially expressed genes (No./%) | Pathway ID |
| --- | --- | --- |
| Purine metabolism | 260 (5.19%) | map00230 |
| Biosynthesis of antibiotics | 255 (5.09%) | map01130 |
| Thiamine metabolism | 213 (4.25%) | map00730 |
| Starch and sucrose metabolism | 146 (2.91%) | map00500 |
| Aminobenzoate degradation | 131 (2.61%) | map00627 |
| Glycolysis / Gluconeogenesis | 90 (1.80%) | map00010 |
| T cell receptor signaling pathway | 77 (1.54%) | map04660 |
| Galactose metabolism | 70 (1.40%) | map00052 |
| Methane metabolism | 69 (1.38%) | map00680 |
| Glyoxylate and dicarboxylate metabolism | 67 (1.34%) | map00630 |
| Carbon fixation in photosynthetic organisms | 65 (1.30%) | map00710 |
| Phenylpropanoid biosynthesis | 59 (1.18%) | map00940 |
| Pyruvate metabolism | 57 (1.14%) | map00620 |
| Glycerolipid metabolism | 52 (1.04%) | map00561 |
| Amino sugar and nucleotide sugar metabolism | 51 (1.02%) | map00520 |
| Fructose and mannose metabolism | 51 (1.02%) | map00051 |
| Glycine, serine and threonine metabolism | 50 (1.0%) | map00260 |
| Pentose phosphate pathway | 49 (0.98%) | map00030 |
| Drug metabolism - other enzymes | 46 (0.92%) | map00983 |
| Pyrimidine metabolism | 43 (0.86%) | map00240 |
| Carbon fixation pathways in prokaryotes | 36 (0.72%) | map00720 |
| Valine, leucine and isoleucine degradation | 35 (0.70%) | map00280 |
| Glutathione metabolism | 34 (0.68%) | map00480 |
| Cyanoamino acid metabolism | 34 (0.68%) | map00460 |
| Tryptophan metabolism | 30 (0.60%) | map00380 |
| Glycerophospholipid metabolism | 30 (0.60%) | map00564 |
| Fatty acid degradation | 29 (0.58%) | map00071 |
| alpha-Linolenic acid metabolism | 27 (0.54%) | map00592 |
| Metabolism of xenobiotics by cytochrome P450 | 27 (0.54%) | map00980 |
| Phenylalanine metabolism | 27 (0.54%) | map00360 |
| beta-Alanine metabolism | 26 (0.52%) | map00410 |
| Phosphatidylinositol signaling system | 26 (0.52%) | map04070 |
| Oxidative phosphorylation | 25 (0.50%) | map00190 |
| Pentose and glucuronate interconversions | 25 (0.50%) | map00040 |
| Ubiquinone and other terpenoid-quinone biosynthesis | 24 (0.48%) | map00130 |
| Arginine and proline metabolism | 23 (0.46%) | map00330 |
| Nitrogen metabolism | 23 (0.46%) | map00910 |
| Terpenoid backbone biosynthesis | 23 (0.46%) | map00900 |
| Cysteine and methionine metabolism | 23 (0.46%) | map00270 |
| Alanine, aspartate and glutamate metabolism | 23 (0.46%) | map00250 |
| Propanoate metabolism | 23 (0.46%) | map00640 |
| Ascorbate and aldarate metabolism | 23 (0.46%) | map00053 |
| Citrate cycle (TCA cycle) | 22 (0.44%) | map00020 |
| Butanoate metabolism | 22 (0.44%) | map00650 |
| Lysine degradation | 21 (0.42%) | map00310 |
| Sphingolipid metabolism | 21 (0.42%) | map00600 |
| Tyrosine metabolism | 21 (0.42%) | map00350 |
| Linoleic acid metabolism | 20 (0.40%) | map00591 |
| Inositol phosphate metabolism | 20 (0.40%) | map00562 |
| Porphyrin and chlorophyll metabolism | 19 (0.38%) | map00860 |
| Sulfur metabolism | 18 (0.36%) | map00920 |
| Retinol metabolism | 18 (0.36%) | map00830 |
| Pantothenate and CoA biosynthesis | 17 (0.34%) | map00770 |
| Phenylalanine, tyrosine and tryptophan biosynthesis | 17 (0.34%) | map00400 |
| Chloroalkane and chloroalkene degradation | 16 (0.32%) | map00625 |
| Other glycan degradation | 16 (0.32%) | map00511 |
| Arachidonic acid metabolism | 15 (0.30%) | map00590 |
| Aminoacyl-tRNA biosynthesis | 15 (0.30%) | map00970 |
| Arginine biosynthesis | 13 (0.26%) | map00220 |
| Caprolactam degradation | 13 (0.26%) | map00930 |
| Histidine metabolism | 13 (0.26%) | map00340 |
| Tropane, piperidine and pyridine alkaloid biosynthesis | 12 (0.24%) | map00960 |
| Ether lipid metabolism | 12 (0.24%) | map00565 |
| Steroid hormone biosynthesis | 12 (0.24%) | map00140 |
| One carbon pool by folate | 11 (0.22%) | map00670 |
| Limonene and pinene degradation | 11 (0.22%) | map00903 |
| Biosynthesis of unsaturated fatty acids | 10 (0.20%) | map01040 |
| Nicotinate and nicotinamide metabolism | 10 (0.20%) | map00760 |
| Selenocompound metabolism | 10 (0.20%) | map00450 |
| Valine, leucine and isoleucine biosynthesis | 9 (0.18%) | map00290 |
| Streptomycin biosynthesis | 9 (0.18%) | map00521 |
| Lysine biosynthesis | 9 (0.18%) | map00300 |
| Isoquinoline alkaloid biosynthesis | 9 (0.18%) | map00950 |
| Fatty acid elongation | 9 (0.18%) | map00062 |
| Plant-pathogen interaction | 8 (0.16%) | map04626 |
| Geraniol degradation | 8 (0.16%) | map00281 |
| Glycosphingolipid biosynthesis - globo series | 8 (0.16%) | map00603 |
| Benzoate degradation | 8 (0.16%) | map00362 |
| Vitamin B6 metabolism | 8 (0.16%) | map00750 |
| Steroid biosynthesis | 8 (0.16%) | map00100 |
| Flavonoid biosynthesis | 8 (0.16%) | map00941 |
| Steroid degradation | 7 (0.14%) | map00984 |
| Caffeine metabolism | 7 (0.14%) | map00232 |
| Toluene degradation | 7 (0.14%) | map00623 |
| Photosynthesis | 7 (0.14%) | map00195 |
| N-Glycan biosynthesis | 7 (0.14%) | map00510 |
| Cutin, suberine and wax biosynthesis | 7 (0.14%) | map00073 |
| Novobiocin biosynthesis | 7 (0.14%) | map00401 |
| Various types of N-glycan biosynthesis | 6 (0.12%) | map00513 |
| Carotenoid biosynthesis | 6 (0.12%) | map00906 |
| Chlorocyclohexane and chlorobenzene degradation | 6 (0.12%) | map00361 |
| Monobactam biosynthesis | 6 (0.12%) | map00261 |
| Monobactam biosynthesis | 6 (0.12%) | map00261 |
| Styrene degradation | 6 (0.12%) | map00643 |
| Glycosaminoglycan biosynthesis - heparan sulfate / heparin | 6 (0.12%) | map00534 |
| Glycosaminoglycan degradation | 6 (0.12%) | map00531 |
| Xylene degradation | 5 (0.10%) | map00622 |
| Glucosinolate biosynthesis | 5 (0.10%) | map00966 |
| Glycosphingolipid biosynthesis - ganglio series | 5 (0.10%) | map00604 |
| Synthesis and degradation of ketone bodies | 5 (0.10%) | map00072 |
| Taurine and hypotaurine metabolism | 4 (0.08%) | map00430 |
| Biotin metabolism | 4 (0.08%) | map00780 |
| Butirosin and neomycin biosynthesis | 4 (0.08%) | map00524 |
| C5-Branched dibasic acid metabolism | 4 (0.08%) | map00660 |
| Carbapenem biosynthesis | 3 (0.06%) | map00332 |
| Naphthalene degradation | 3 (0.06%) | map00626 |
| Zeatin biosynthesis | 3 (0.06%) | map00908 |
| Indole alkaloid biosynthesis | 3 (0.06%) | map00901 |
| Benzoxazinoid biosynthesis | 3 (0.06%) | map00402 |
| Isoflavonoid biosynthesis | 3 (0.06%) | map00943 |
| Riboflavin metabolism | 3 (0.06%) | map00740 |
| Ethylbenzene degradation | 3 (0.06%) | map00642 |
| Folate biosynthesis | 3 (0.06%) | map00790 |
| Insect hormone biosynthesis | 2 (0.04%) | map00981 |
| Lipoic acid metabolism | 2 (0.04%) | map00785 |
| Primary bile acid biosynthesis | 2 (0.04%) | map00120 |
| Sesquiterpenoid and triterpenoid biosynthesis | 2 (0.04%) | map00909 |
| Diterpenoid biosynthesis | 2 (0.04%) | map00904 |
| Fluorobenzoate degradation | 2 (0.04%) | map00364 |
| Stilbenoid, diarylheptanoid and gingerol biosynthesis | 2 (0.04%) | map00945 |
| Biosynthesis of terpenoids and steroids | 2 (0.04%) | map01062 |
| Aflatoxin biosynthesis | 2 (0.04%) | map00254 |
| Tetracycline biosynthesis | 2 (0.04%) | map00253 |
| Phosphonate and phosphinate metabolism | 2 (0.04%) | map00440 |
| mTOR signaling pathway | 2 (0.04%) | map04150 |
| Polyketide sugar unit biosynthesis | 1 (0.02%) | map00523 |
| Other types of O-glycan biosynthesis | 1 (0.02%) | map00514 |
| D-Alanine metabolism | 1 (0.02%) | map00473 |
| D-Glutamine and D-glutamate metabolism | 1 (0.02%) | map00471 |
| Peptidoglycan biosynthesis | 1 (0.02%) | map00550 |
| Flavone and flavonol biosynthesis | 1 (0.02%) | map00944 |
| Biosynthesis of vancomycin group antibiotics | 1 (0.02%) | map01055 |
| Biosynthesis of ansamycins | 1 (0.02%) | map01051 |
| Atrazine degradation | 1 (0.02%) | map00791 |

Supplementary Table S3. Real-time PCR Primers for quantification of gene expression.

| Target gene | Primer name | sequence (5´-3´) | Reference |
| --- | --- | --- | --- |
| PR-5 | PR-5F | GCAGCTGAACAGCGGCGAGACGTGGAAC |  |
|  | PR-5R | GCCGGTGCTGCAGGAGAAGCCCATGC |  |
| PR-1 | PR1-F2 | AGCACAAGGCTGCAGTCGTA | This study |
|  | PR1-R2 | CTTGCAGTCGCCGATCCT |  |
| Thaumatin-like PR3 | Thi-F2 | AACTGCCCGGATGCCTATC | This study |
|  | Thi-R2 | CGTTGCAGCCGTGTGTTTT |  |
| Chitinase 5 | Chi5-F1 | CCAGTGGTGGCGTTCAAGA | This study |
|  | Chi5-R1 | CACCCCATGCACGTTGGT |  |
| Chitinase 2 | Chi2-F2 | CCCGATGTTGAACGACTTCTG | This study |
|  | Chi2-R2 | TCGGTTTCTACAAGCGCTACTG |  |
| WRKY | WRKY-F1 | CGCTCCACCCTCTTCTTCAC | This study |
|  | WRKY-R1 | TGTGCTGTGTGCAGGAACTACTAC |  |
| LpGAPDH | LpGAPDH-F | CATCACCATTGTCTCCAACG |  |
|  | LpGAPDH-R | AACCTTCAACGATGCCAAAC |  |

Supplementary Table S4. Sequences of the genes under GO terms significantly enriched in the resistant (R) genotype.

Supplementary Table S5. Sequences of differentially expressed genes that can be considered as potential candidate genes involved in response to *M. nivale* in two Lolium perenne , cv. Fagerlin genotypes, R (resistant genotype), and S (susceptible genotype).
